# A holistic and proactive approach to forecasting cyber threats

**DOI:** 10.1038/s41598-023-35198-1

**Published:** 2023-05-17

**Authors:** Zaid Almahmoud, Paul D. Yoo, Omar Alhussein, Ilyas Farhat, Ernesto Damiani

**Affiliations:** 1 Department of Computer Science and Information Systems, University of London, Birkbeck College, London, United Kingdom; 2 Huawei Technologies Canada, Ottawa, Canada; 3 Department of Electrical and Computer Engineering, University of Waterloo, Waterloo, Canada; 4 Department of Computer Science, Università degli Studi di Milano, Milan, Italy; 5 Center for Cyber-Physical Systems (C2PS), Khalifa University, Abu Dhabi, United Arab Emirates

**Keywords:** Computer science, Information technology

## Abstract

Traditionally, cyber-attack detection relies on reactive, assistive techniques, where pattern-matching algorithms help human experts to scan system logs and network traffic for known virus or malware signatures. Recent research has introduced effective Machine Learning (ML) models for cyber-attack detection, promising to automate the task of detecting, tracking and blocking malware and intruders. Much less effort has been devoted to cyber-attack prediction, especially beyond the short-term time scale of hours and days. Approaches that can forecast attacks likely to happen in the longer term are desirable, as this gives defenders more time to develop and share defensive actions and tools. Today, long-term predictions of attack waves are mostly based on the subjective perceptiveness of experienced human experts, which can be impaired by the scarcity of cyber-security expertise. This paper introduces a novel ML-based approach that leverages unstructured big data and logs to forecast the trend of cyber-attacks at a large scale, years in advance. To this end, we put forward a framework that utilises a monthly dataset of major cyber incidents in 36 countries over the past 11 years, with new features extracted from three major categories of big data sources, namely the scientific research literature, news, blogs, and tweets. Our framework not only identifies future attack trends in an automated fashion, but also generates a threat cycle that drills down into five key phases that constitute the life cycle of all 42 known cyber threats.

## Introduction

Running a global technology infrastructure in an increasingly de-globalised world raises unprecedented security issues. In the past decade, we have witnessed waves of cyber-attacks that caused major damage to governments, organisations and enterprises, affecting their bottom lines^1^. Nevertheless, cyber-defences remained *reactive* in nature, involving significant overhead in terms of execution time. This latency is due to the complex pattern-matching operations required to identify the signatures of *polymorphic* malware^2^, which shows different behaviour each time it is run. More recently, ML-based models were introduced relying on *anomaly detection* algorithms. Although these models have shown a good capability to detect unknown attacks, they may classify benign behaviour as abnormal^3^, giving rise to a false alarm.

We argue that data availability can enable a *proactive* defense, acting before a potential threat escalates into an actual incident. Concerning non-cyber threats, including terrorism and military attacks, proactive approaches alleviate, delay, and even prevent incidents from arising in the first place. Massive software programs are available to assess the intention, potential damages, attack methods, and alternative options for a terrorist attack^4^. We claim that cyber-attacks should be no exception, and that nowadays we have the capabilities to carry out proactive, low latency cyber-defenses based on ML^5^.

Indeed, ML models can provide accurate and reliable forecasts. For example, ML models such as AlphaFold2^6^ and RoseTTAFold^7^ can predict a protein’s three-dimensional structure from its linear sequence. Cyber-security data, however, poses its unique challenges. Cyber-incidents are highly sensitive events and are usually kept confidential since they affect the involved organisations’ reputation. It is often difficult to keep track of these incidents, because they can go unnoticed even by the victim. It is also worth mentioning that pre-processing cyber-security data is challenging, due to characteristics such as lack of structure, diversity in format, and high rates of missing values which distort the findings.

When devising a ML-based method, one can rely on manual feature identification and engineering, or try and learn the features from raw data. In the context of cyber-incidents, there are many factors (*i.e.*, potential features) that could lead to the occurrence of an attack. Wars and political conflicts between countries often lead to cyber-warfare^8,9^. The number of mentions of a certain attack appearing in scientific articles may correlate well with the actual incident rate. Also, cyber-attacks often take place on holidays, anniversaries and other politically significant dates^5^. Finding the right features out of unstructured big data is one of the key strands of our proposed framework.

The remainder of the paper is structured as follows. The “Literature review” section presents an overview of the related work and highlights the research gaps and our contributions. The “Methods” section describes the framework design, including the construction of the dataset and the building of the model. The “Results” section presents the validation results of our model, the trend analysis and forecast, and a detailed description of the developed threat cycle. Lastly, the “Discussion” section offers a critical evaluation of our work, highlighting its strengths and limitations, and provides recommendations for future research.

## Literature review

In recent years, the literature has extensively covered different cyber threats across various application domains, and researchers have proposed several solutions to mitigate these threats. In the Social Internet of Vehicles (SIoV), one of the primary concerns is the interception and tampering of sensitive information by attackers^10^. To address this, a secure authentication protocol has been proposed that utilises confidential computing environments to ensure the privacy of vehicle-generated data. Another application domain that has been studied is the privacy of image data, specifically lane images in rural areas^11^. The proposed methodology uses Error Level Analysis (ELA) and artificial neural network (ANN) algorithms to classify lane images as genuine or fake, with the U-Net model for lane detection in bona fide images. The final images are secured using the proxy re-encryption technique with RSA and ECC algorithms, and maintained using fog computing to protect against forgery.

Another application domain that has been studied is the security of Wireless Mesh Networks (WMNs) in the context of the Internet of Things (IoT)^12^. WMNs rely on cooperative forwarding, making them vulnerable to various attacks, including packet drop/modification, badmouthing, on-off, and collusion attacks. To address this, a novel trust mechanism framework has been proposed that differentiates between legitimate and malicious nodes using direct and indirect trust computation. The framework utilises a two-hop mechanism to observe the packet forwarding behaviour of neighbours, and a weighted D-S theory to aggregate recommendations from different nodes. While these solutions have shown promising results in addressing cyber threats, it is important to anticipate the type of threat that may arise to ensure that the solutions can be effectively deployed. By proactively identifying and anticipating cyber threats, organisations can better prepare themselves to protect their systems and data from potential attacks.

While we are relatively successful in detecting and classifying cyber-attacks when they occur^13–15^, there has been a much more limited success in predicting them. Some studies exist on short-term predictive capability^16–26^, such as predicting the number or source of attacks to be expected in the next hours or days. The majority of this work performs the prediction in restricted settings (*e.g.*, against a specific entity or organisation) where historical data are available^18,19,25^. Forecasting attack occurrences has been attempted by using statistical methods, especially when parametric data distributions could be assumed^16,17^, as well as by using ML models^20^. Other methods adopt a Bayesian setting and build *event graphs* suitable for estimating the conditional probability of an attack following a given chain of events^21^. Such techniques rely on libraries of predefined attack graphs: they can identify the known attack most likely to happen, but are helpless against never-experienced-before, *zero-day* attacks.

Other approaches try to identify potential attackers by using network entity reputation and scoring^26^. A small but growing body of research explores the fusion of heterogeneous features (warning signals) to forecast cyber-threats using ML. Warning signs may include the number of mentions of a victim organisation on Twitter^18^, mentions in news articles about the victim entity^19^, and digital traces from dark web hacker forums^20^. Our literature review is summarised in Table 1.

Forecasting the cyber-threats that will most likely turn into attacks in the medium and long term is of significant importance. It not only gives to cyber-security agencies the time to evaluate the existing defence measures, but also assists them in identifying areas where to develop preventive solutions. Long-term prediction of cyber-threats, however, still relies on the subjective perceptions of human security experts^27,28^. Unlike a fully automated procedure based on quantitative metrics, the human-based approach is prone to bias based on scientific or technical interests^29^. Also, quantitative predictions are crucial to scientific objectivity^30^. In summary, we highlight the following research gaps:

- Current research primarily focuses on detecting (*i.e.*, reactive) rather than predicting cyber-attacks (*i.e.*, proactive).
- Available predictive methods for cyber-attacks are mostly limited to short-term predictions.
- Current predictive methods for cyber-attacks are limited to restricted settings (*e.g.*, a particular network or system).
- Long-term prediction of cyber-attacks is currently performed by human experts, whose judgement is subjective and prone to bias and disagreement.

### Research contributions

Our objective is to fill these research gaps by a proactive, long-term, and holistic approach to attack prediction. The proposed framework gives cyber-security agencies sufficient time to evaluate existing defence measures while also providing objective and accurate representation of the forecast. Our study is aimed at predicting the trend of cyber-attacks up to three years in advance, utilising big data sources and ML techniques. Our ML models are learned from heterogeneous features extracted from massive, unstructured data sources, namely, Hackmageddon^9^, Elsevier^31^, Twitter^32^, and Python APIs^33^. Hackmageddon provides more than 15, 000 records of global cyber-incidents since the year 2011, while Elsevier API offers access to the Scopus database, the largest abstract and citation database of peer-reviewed literature with over 27,000,000 documents^34^. The number of relevant tweets we collected is around 9 million. Our study covers 36 countries and 42 major attack types. The proposed framework not only provides the forecast and categorisation of the threats, but also generates a threat life-cycle model, whose the five key phases underlie the life cycle of all 42 known cyber-threats. The key contribution of this study consists of the following:

- A novel dataset is constructed using big unstructured data (*i.e.*, Hackmageddon) including news and government advisories, in addition to Elsevier, Twitter, and Python API. The dataset comprises monthly counts of cyber-attacks and other unique features, covering 42 attack types across 36 countries.
- Our proactive approach offers long-term forecasting by predicting threats up to 3 years in advance.
- Our approach is holistic in nature, as it does not limit itself to specific entities or regions. Instead, it provides projections of attacks across 36 countries situated in diverse parts of the world.
- Our approach is completely automated and quantitative, effectively addressing the issue of bias in human predictions and providing a precise forecast.
- By analysing past and predicted future data, we have classified threats into four main groups and provided a forecast of 42 attacks until 2025.
- The first threat cycle is proposed, which delineates the distinct phases in the life cycle of 42 cyber-attack types.

## Methods

### The framework of forecasting cyber threats

**Figure 1:**
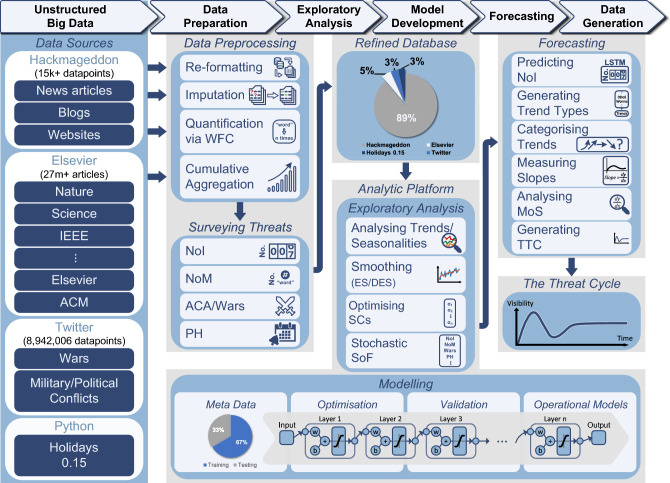
The workflow and architecture of forecasting cyber threats. The ground truth of Number of Incidents (NoI) was extracted from Hackmageddon which has over 15,000 daily records of cyber incidents worldwide over the past 11 years. Additional features were obtained including the Number of Mentions (NoM) of each attack in the scientific literature using Elsevier API which gives access to over 27 million documents. The number of tweets about Armed Conflict Areas/Wars (ACA) was also obtained using Twitter API for each country, with a total of approximately 9 million tweets. Finally, the number of Public Holidays (PH) in each country was obtained using the holidays library in Python. The data preparation phase includes data re-formatting, imputation and quantification using Word Frequency Counter (WFC) to obtain the monthly occurrence of attacks per country and Cumulative Aggregation (CA) to obtain the sum for all countries. The monthly NoM, ACA and PHs were quantified and aggregated using CA. The numerical features were then combined and stored in the refined database. The percentages in the refined database are based on the contribution of each data source. In the exploratory analysis phase, the analytic platform analyses the trend and performs data smoothing using Exponential Smoothing (ES), Double Exponential Smoothing (DES) and No Smoothing (NS). The smoothing methods and Smoothing Constants (SCs) were chosen for each attack followed by the Stochastic Selection of Features (SoF). In the model development phase, the meta data was partitioned into approximately 67% for training and 33% for testing. The models were learned using the encoder-decoder architecture of the Bayesian Long Short-Term Memory (B-LSTM). The optimisation component finds the set of hyper-parameters that minimises the error (i.e., M-SMAPE), which is then used for learning the operational models. In the forecasting phase, we used the operational models to predict the next three years’ NoIs. Analysing the predicted data, trend types were identified and attacks were categorised into four different trends. The slope of each attack was then measured and the Magnitude of Slope (MoS) was analysed. The final output is The Threat Cycle (TTC) illustrating the attacks trend, status, and direction in the next 3 years.

The architecture of our framework for forecasting cyber threats is illustrated in Fig. 1. As seen in the Data Sources component (l.h.s), to harness all the relevant data and extract meaningful insights, our framework utilises various sources of unstructured data. One of our main sources is Hackmageddon, which includes massive textual data on major cyber-attacks (approx. 15,334 incidents) dating back to July 2011. We refer to the monthly number of attacks in the list as the *Number of Incidents* (NoI). Also, Elsevier’s Application Programming Interface (API) gives access to a very large corpus of scientific articles and data sets from thousands of sources. Utilising this API, we obtained the *Number of Mentions* (NoM) (*e.g.*, monthly) of each attack that appeared in the scientific publications. This NoM data is of particular importance as it can be used as the ground truth for attack types that do not appear in Hackmageddon. During the preliminary research phase, we examined all the potentially relevant features and noticed that wars/political conflicts are highly correlated to the number of cyber-events. These data were then extracted via Twitter API as Armed Conflict Areas/Wars (ACA). Lastly, as attacks often take place around holidays, Python’s holidays package was used to obtain the number of public holidays per month for each country, which is referred to as *Public Holidays *(PH).

To ensure the accuracy and quality of Hackmageddon data, we validated it using the statistics from official sources across government, academia, research institutes and technology organisations. For a ransomware example, the Cybersecurity & Infrastructure Security Agency stated in their 2021 trend report that cybersecurity authorities in the United States, Australia, and the United Kingdom observed an increase in sophisticated, high-impact ransomware incidents against critical infrastructure organisations globally^35^. The WannaCry attack in the dataset was also validated with Ghafur *et al*’s^1^ statement in their article: “WannaCry ransomware attack was a global epidemic that took place in May 2017”.

An example of an entry in the Hackmageddon dataset is shown in Table 2. Each entry includes the incident date, the description of the attack, the attack type, and the target country. Data pre-processing (Fig. 1) focused on noise reduction through imputing missing values (*e.g.*, countries), which were often observed in the earlier years. We were able to impute these values from the description column or occasionally, by looking up the entity location using Google.

The textual data were quantified via our Word Frequency Counter (WFC), which counted the number of each attack type per month as in Table 3. Cumulative Aggregation (CA) obtained the number of attacks for all countries combined and an example of a data entry after transformation includes the month, and the number of attacks against each country (and all countries combined) for each attack type. By adding features such as NoM, ACA, and PH, we ended up having additional features that we appended to the dataset as shown in Table 4. Our final dataset covers 42 common types of attacks in 36 countries. The full list of attacks is provided in Table 5. The list of the countries is given in Supplementary Table S1.

To analyse and investigate the main characteristics of our data, an exploratory analysis was conducted focusing on the visualisation and identification of key patterns such as trend and seasonality, correlated features, missing data and outliers. For seasonal data, we smoothed out the seasonality so that we could identify the trend while removing the noise in the time series^36^. The smoothing type and constants were optimised along with the ML model (see Optimisation for details). We applied Stochastic selection of Features (SoF) to find the subset of features that minimises the prediction error, and compared the univariate against the multivariate approach.

For the modelling, we built a Bayesian encoder-decoder Long Short-Term Memory (B-LSTM) network. B-LSTM models have been proposed to predict “perfect wave” events like the onset of stock market “bear” periods on the basis of multiple warning signs, each having different time dynamics^37^. Encoder-decoder architectures can manage inputs and outputs that both consist of variable-length sequences. The encoder stage encodes a sequence into a fixed-length vector representation (known as the *latent* representation). The decoder prompts the latent representation to predict a sequence. By applying an efficient latent representation, we train the model to consider all the useful warning information from the input sequence - regardless of its position - and disregard the noise.

Our Bayesian variation of the encoder-decoder LSTM network considers the weights of the model as random variables. This way, we extract epistemic uncertainty via (approximate) Bayesian inference, which quantifies the prediction error due to insufficient information^38^. This is an important parameter, as epistemic uncertainty can be reduced by better intelligence, *i.e.*, by acquiring more samples and new informative features. Details are provided in “Bayesian long short-term memory” section.

Our overall analytical platform learns an operational model for each attack type. Here, we evaluated the model’s performance in predicting the threat trend 36 months in advance. A newly modified symmetric Mean Absolute Percentage Error (M-SMAPE) was devised as the evaluation metric, where we added a penalty term that accounts for the trend direction. More details are provided in the “Evaluation metrics” section.

### Feature extraction

Below, we provide the details of the process that transforms raw data into numerical features, obtaining the ground truth NoI and the additional features NoM, ACA and PH.

- NoI: The number of daily incidents in Hackmageddon was transformed from the purely unstructured daily description of attacks along with the attack and country columns, to the monthly count of incidents for each attack in each country. Within the description, multiple related attacks may appear, which are not necessarily in the attack column. Let $E_{x_i}$ denote the set of entries during the month $x_i$ in Hackmageddon dataset. Let $a_j$ and $c_k$ denote the *j*^th^ attack and *k*^th^ country. Then NoI can be expressed as follows:
  1 $$\begin{aligned} NoI(x_i,a_j,c_k) = \sum _{e \in E_{x_i}} Z(a_j,c_k,e) \end{aligned}$$
  where $Z(a_j,c_k,e)$ is a function that evaluates to 1 if $a_j$ appears either in the description or in the attack columns of entry *e* and $c_k$ appears in the country column of *e*. Otherwise, the function evaluates to 0. Next, we performed CA to obtain the monthly count of attacks in all countries combined for each attack type as follows:
  2 $$\begin{aligned} NoI(x_i,a_j) = \sum _{k=0}^{K} NoI(x_i,a_j,c_k) \end{aligned}$$
- NoM: We wrote a Python script to query Elsevier API for the number of mentions of each attack during each month^31^. The search covers the title, abstract and keywords of published research papers that are stored in Scopus database^39^. Let $P_{x_i}$ denote the set of research papers in Scopus published during the month $x_i$. Also, let $W_{p}$ denote the set of words in the title, abstract and keywords of research paper *p*. Then NoM can be expressed as follows:
  3 $$\begin{aligned} NoM(x_i,a_j) = \sum _{p \in P_{x_i}} \sum _{w \in W_{p}} U(w,a_j) \end{aligned}$$
  where $U(w,a_j)$ evaluates to 1 if $w=a_j$, and to 0 otherwise.
- ACA: Using Twitter API in Python^32^, we wrote a query to obtain the number of tweets with keywords related to political conflicts or military attacks associated with each country during each month. The keywords used for each country are summarised in Supplementary Table S2, representing our query. Formally, let $T_{x_i}$ denote the set of all tweets during the month $x_i$. Then ACA can be expressed as follows:
  4 $$\begin{aligned} ACA(x_{i}, c_{k})= \sum _{t \in T_{x_i}} Q(t,c_k) \end{aligned}$$
  where $Q(t,c_k)$ evaluates to 1 if the query in Supplementary Table S2 evaluates to 1 given *t* and $c_k$. Otherwise, it evaluates to 0.

- PH: We used the Python holidays library^33^ to count the number of days that are considered public holidays in each country during each month. More formally, this can be expressed as follows:
  5 $$\begin{aligned} PH(x_{i}, c_{k})= \sum _{d \in x_{i}} H(d,c_k) \end{aligned}$$
  where $H(d,c_k)$ evaluates to 1 if the day *d* in the country $c_k$ is a public holiday, and to 0 otherwise. In (4) and (5), CA was used to obtain the count for all countries combined as in (2).

### Data integration

Based on Eqs. (1)–(5), we obtain the following columns for each month:

- NoI_C: The number of incidents for each attack type in each country ($42 \times 36$ columns) [Hackmageddon].
- NoI: The total number of incidents for each attack type (42 columns) [Hackmageddon].
- NoM: The number of mentions of each attack type in research articles (42 columns) [Elsevier].
- ACA_C: The number of tweets about wars and conflicts related to each country (36 columns) [Twitter].
- ACA: The total number of tweets about wars and conflicts (1 column) [Twitter].
- PH_C: The number of public holidays in each country (36 columns) [Python].
- PH: The total number of public holidays (1 column) [Python].

In the aforementioned list of columns, the name enclosed within square brackets denotes the source of data. By matching and combining these columns, we derive our monthly dataset, wherein each row represents a distinct month. A concrete example can be found in Tables 3 and 4, which, taken together, constitute a single observation in our dataset. The dataset can be expanded through the inclusion of other monthly features as supplementary columns. Additionally, the dataset may be augmented with further samples as additional monthly records become available. Some suggestions for extending the dataset are provided in the “Discussion” section.

### Data smoothing

We tested multiple smoothing methods and selected the one that resulted in the model with the lowest M-SMAPE during the hyper-parameter optimisation process. The methods we tested include exponential smoothing (ES), double exponential smoothing (DES) and no smoothing (NS). Let $\alpha$ be the smoothing constant. Then the ES formula is:

6 $$\begin{aligned} S(x_{i})= {\left\{ \begin{array}{ll} \alpha D(x_{i}) + (1-\alpha ) S(x_{i-1}),&{} \text {if } i\ge 1\\ D(x_{0}), &{} \text {otherwise} \end{array}\right. } \end{aligned}$$

where $D(x_{i})$ denotes the original data at month $x_{i}$. For the DES formula, let $\alpha$ and $\beta$ be the smoothing constants. We first define the level $l(x_{i})$ and the trend $\tau (x_{i})$ as follows:

7 $$\begin{aligned}{} & {} l(x_{i})= {\left\{ \begin{array}{ll} \alpha D(x_{i}) + (1-\alpha ) (l(x_{i-1})+\tau (x_{i-1})),&{} \text {if } i\ge 1\\ D(x_{0}), &{} \text {otherwise} \end{array}\right. } \end{aligned}$$

8 $$\begin{aligned}{} & {} \quad \tau (x_{i})= {\left\{ \begin{array}{ll} \beta (l(x_{i})-l(x_{i-1})) + (1-\beta )\tau (x_{i-1}),&{} \text {if } i\ge 1\\ D(x_{1})-D(x_{0}), &{} \text {otherwise} \end{array}\right. } \end{aligned}$$

then, DES is expressed as follows:

9 $$\begin{aligned} DS(x_{i})= {\left\{ \begin{array}{ll} l(x_{i})+\tau (x_{i}),&{} \text {if } i\ge 1\\ D(x_{0}), &{} \text {otherwise} \end{array}\right. } \end{aligned}$$

The smoothing constants ($\alpha$ and $\beta$) in the aforementioned methods are chosen as the predictive results of the ML model that gives the lowest M-SMAPE during the hyper-parameter optimisation process. Supplementary Fig. S5 depicts an example for the DES result.

### Bayesian long short-term memory

LSTM is a type of recurrent neural network (RNN) that uses lagged observations to forecast the future time steps^30^. It was introduced as a solution to the so-called *vanishing/exploding* gradient problem of traditional RNNs^40^, where the partial derivative of the loss function may suddenly approach zero at some point of the training. In LSTM, the input is passed to the network cell, which combines it with the hidden state and cell state values from previous time steps to produce the next states. The hidden state can be thought of as a short-term memory since it stores information from recent periods in a weighted manner. On the other hand, the cell state is meant to remember all the past information from previous intervals and store them in the LSTM cell. The cell state thus represents the long-term memory.

LSTM networks are well-suited for time-series forecasting, due to their proficiency in retaining both long-term and short-term temporal dependencies^41,42^. By leveraging their ability to capture these dependencies within cyber-attack data, LSTM networks can effectively recognise recurring patterns in the attack time-series. Moreover, the LSTM model is capable of learning intricate temporal patterns in the data and can uncover inter-correlations between various variables, making it a compelling option for multivariate time-series analysis^43^.

Given a sequence of LSTM cells, each processing a single time-step from the past, the final hidden state is encoded into a fixed-length vector. Then, a decoder uses this vector to forecast future values. Using such architecture, we can map a sequence of time steps to another sequence of time steps, where the number of steps in each sequence can be set as needed. This technique is referred to as *encoder-decoder* architecture.

Because we have relatively short sequences within our refined data (*e.g.*, 129 monthly data points over the period from July 2011 to March 2022), it is crucial to extract the source of uncertainty, known as *epistemic* uncertainty^44^, which is caused by lack of knowledge. In principle, epistemic uncertainty can be reduced with more knowledge either in the form of new features or more samples. Deterministic (non-stochastic) neural network models are not adequate to this task as they provide point estimates of model parameters. Rather, we utilise a Bayesian framework to capture epistemic uncertainty. Namely, we adopt the Monte Carlo dropout method proposed by Gal *et al.*^45^, who showed that the use of non-random dropout neurons during ML training (and inference) provides a Bayesian approximation of the deep Gaussian processes. Specifically, during the training of our LSTM encoder-decoder network, we applied the same dropout mask at every time-step (rather than applying a dropout mask randomly from time-step to time-step). This technique, known as *recurrent dropout * is readily available in Keras^46^. During the inference phase, we run trained model multiple times with recurrent dropout to produce a distribution of predictive results. Such prediction is shown in Fig. 4.

**Figure 2:**
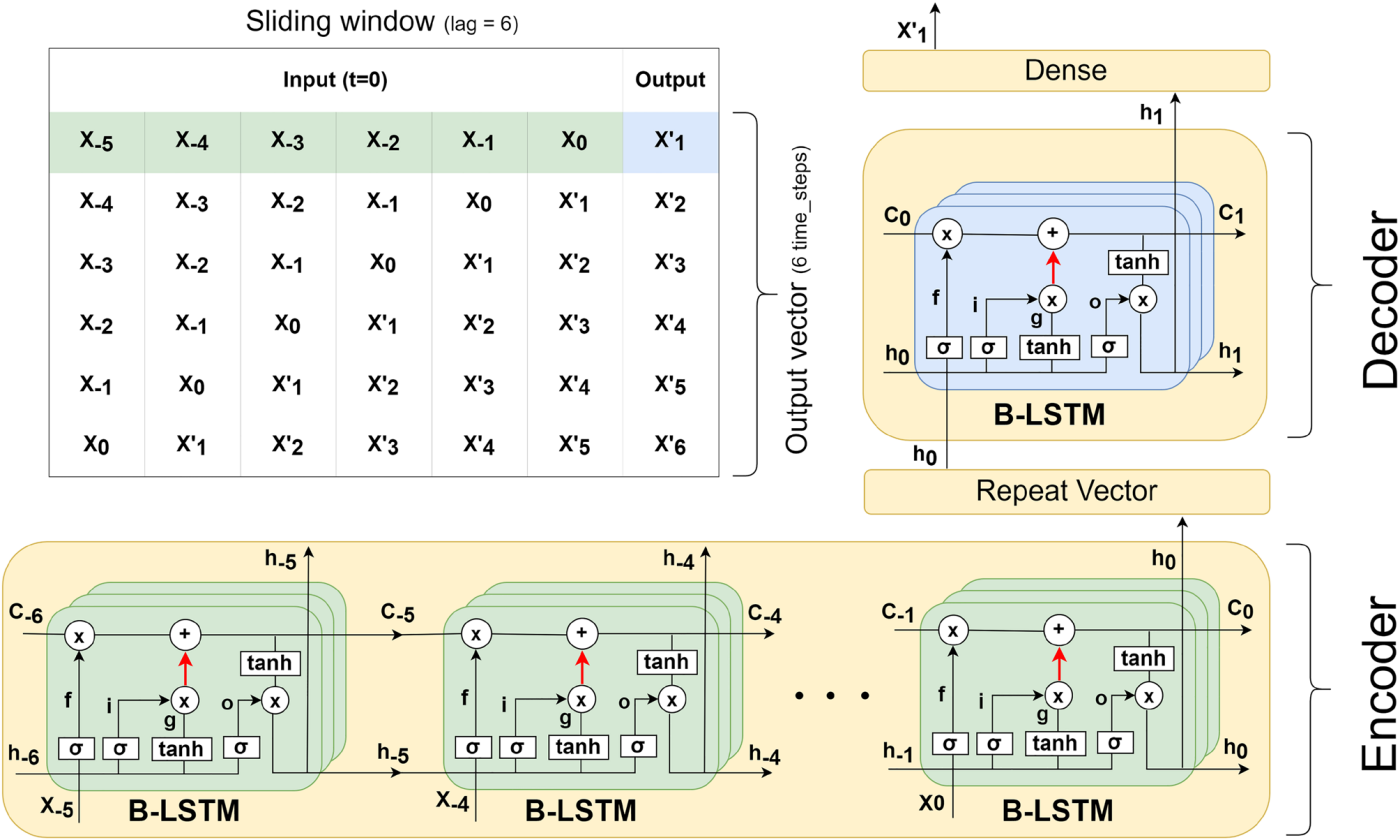
The encoder-decoder architecture of Bayesian Long Short-Term Memory (B-LSTM). $X_{i}$ stands for the input at time-step *i*. $h_{i}$ stands for the hidden state, which stores information from the recent time steps (short-term). $C_{i}$ stands for the cell state, which stores all processed information from the past (long-term). The number of input time steps in the encoder is a variable tuned as a hyper-parameter, while the output in the decoder is a single time-step. The depth and number of layers are another set of hyper-parameters tuned during the model optimisation. The red arrows indicate a recurrent dropout maintained during the testing and prediction. The figure shows an example for an input with time lag=6 and a single layer. The final hidden state $h_{0}$ produced by the encoder is passed to the Repeat Vector layer to convert it from 2 dimensional output to 3 dimensional input as expected by the decoder. The decoder processes the input and produces the final hidden state $h_{1}$. This hidden state is finally passed to a dense layer to produce the output. The table illustrates the concept of sliding window method used to forecast multiple time steps during the testing and prediction (i.e., using the output at a time-step as an input to forecast the next time-step). Using this concept, we can predict as many time steps as needed. In the table, an output vector of 6 time steps was predicted.

Figure 2 shows our encoder-decoder B-LSTM architecture. The hidden state and cell state are denoted respectively by $h_{i}$ and $C_{i}$, while the input is denoted by $X_{i}$. Here, the length of the input sequence (lag) is a hyper-parameter tuned to produce the optimal model, where the output is a single time-step. The number of cells (*i.e.*, the depth of each layer) is tuned as a hyper-parameter in the range between 25 and 200 cells. Moreover, we used one or two layers, tuning the number of layers to each attack type. For the univariate model we used a standard Rectified Linear Unit (ReLU) activation function, while for the multivariate model we used a Leaky ReLU. Standard ReLU computes the function $f(x)=max(0,x)$, thresholding the activation at zero. In the multivariate case, zero-thresholding may generate the same ReLU output for many input vectors, making the model convergence slower^47^. With Leaky ReLU, instead of defining ReLU as zero when $x < 0$, we introduce a negative slope $\alpha =0.2$. Additionally, we used recurrent dropout (*i.e.*, arrows in red as shown in Fig. 2), where the probability of dropping out is another hyper-parameter that we tune as described above, following Gal’s method^48^. The tuned dropout value is maintained during the testing and prediction as previously mentioned. Once the final hidden vector $h_{0}$ is produced by the encoder, the Repeat Vector layer is used as an adapter to reshape it from the bi-dimensional output of the encoder (*e.g.*, $h_{0}$) to the three-dimensional input expected by the decoder. The decoder processes the input and produces the hidden state, which is then passed to a dense layer to produce the final output.

Each time-step corresponds to a month in our model. Since the model is learnt to predict a single time-step (single month), we use a sliding window during the prediction phase to forecast 36 (monthly) data points. In other words, we predict a single month at each step, and the predicted value is fed back for the prediction of the following month. This concept is illustrated in the table shown in Fig. 2. Utilising a single time-step in the model’s output minimises the size of the sliding window, which in turn allows for training with as many observations as possible with such limited data.

The difference between the univariate and multivariate B-LSTMs is that the latter carries additional features in each time-step. Thus, instead of passing a scalar input value to the network, we pass a vector of features including the ground truth at each time-step. The model predicts a vector of features as an output, from which we retrieve the ground truth, and use it along with the other predicted features as an input to predict the next time-step.

### Evaluation metrics

The evaluation metric SMAPE is a percentage (or relative) error based accuracy measure that judges the prediction performance purely on how far the predicted value is from the actual value^49^. It is expressed by the following formula:

10 $$\begin{aligned} SMAPE= \frac{100\%}{n} \sum _{t=1}^{n} \frac{|F_{t} - A_{t} |}{|F_{t}|+ |A_{t} |} \end{aligned}$$

where $F_{t}$ and $A_{t}$ denote the predicted and actual values at time *t*. This metric returns a value between 0% and 100%. Given that our data has zero values in some months (*e.g.*, emerging threats), the issue of division by zero may arise, a problem that often emerges when using standard MAPE (Mean Absolute Percentage Error). We find SMAPE to be resilient to this problem, since it has both the actual and predicted values in the denominator.

Recall that our model aims to predict a curve (corresponding to multiple time steps). Using plain SMAPE as the evaluation metric, the “best” model may turn out to be simply a straight line passing through the same points of the fluctuating actual curve. However, this is undesired in our case since our priority is to predict the trend direction (or slope) over its intensity or value at a certain point. We hence add a penalty term to SMAPE that we apply when the height of the predicted curve is relatively smaller than that of the actual curve. This yields the modified SMAPE (M-SMAPE). More formally, let *I*(*V*) be the height of the curve *V*, calculated as follows:

11 $$\begin{aligned} I (V)= {\max }_{t \in [n]} V_{t} - {\min }_{t \in [n]} V_{t} \end{aligned}$$

where *n* is the curve width or the number of data points. Let *A* and *F* denote the actual and predicted curves. We define M-SMAPE as follows:

12 $$\begin{aligned} MSMAPE= {\left\{ \begin{array}{ll} SMAPE+100\% \gamma ,&{} \text {if } I(F) < I(A)/d\\ SMAPE, &{} \text {otherwise} \end{array}\right. } \end{aligned}$$

where $\gamma$ is a penalty constant between 0 and 1, and *d* is another constant $\ge$ 1. In our experiment, we set $\gamma$ to 0.3, and *d* to 3, as we found these to be reasonable values by trial and error. We note that the range of possible values of M-SMAPE is between 0% and (100 + 100 $\gamma$)% after this modification. By running multiple experiments we found out that the modified evaluation metric is more suitable for our scenario, and therefore was adopted for the model’s evaluation.

### Optimisation

On average, our model was trained on around 67% of the refined data, which is equivalent to approximately 7.2 years. We kept the rest, approximately 33% (3 years + lag period), for validation. These percentages may slightly differ for different attack types depending on the optimal lag period selected.

For hyper-parameter optimisation, we performed a random search with 60 iterations, to obtain the set of features, smoothing methods and constants, and model’s hyper-parameters that results in the model with the lowest M-SMAPE. Random search is a simple and efficient technique for hyper-parameter optimisation, with advantages including efficiency, flexibility, robustness, and scalability. The technique has been studied extensively in the literature and was found to be superior to grid search in many cases^50^. For each set of hyper-parameters, the model was trained using the mean squared error (MSE) as the loss function, and while using ADAM as the optimisation algorithm^51^. Then, the model was validated by forecasting 3 years while using M-SMAPE as the evaluation metric, and the average performance was recorded over 3 different seeds. Once the set of hyper-parameters with the minimum M-SMAPE was obtained, we used it to train the model on the full data, after which we predicted the trend for the next 3 years (until March, 2025).

The first group of hyper-parameters is the subset of features in the case of the multivariate model. Here, we experimented with each of the 3 features separately (NoM, ACA or PH) along with the ground truth (NoI), in addition to the combination of all features. The second group is the smoothing methods and constants. The set of methods includes ES, DES and NS, as previously discussed. The set of values for the smoothing constant $\alpha$ ranges from 0.05 to 0.7 while the set of values for the smoothing constant $\beta$ (for DES) ranges from 0.3 to 0.7. Next is the optimisation of the lag period with values that range from 1 to 12 months. This is followed by the model’s hyper-parameters which include the learning rate with values that range from $6\times 10^{-4}$ to $1\times 10^{-2}$, the number of epochs with values between 30 and 200, the number of layers in the range 1 to 2, the number of units in the range 25 to 200, and the recurrent dropout value between 0.2 and 0.5. The range of these values was obtained from the literature and the online code repositories^52^.

## Results

### Validation and comparative analysis

**Figure 3:**
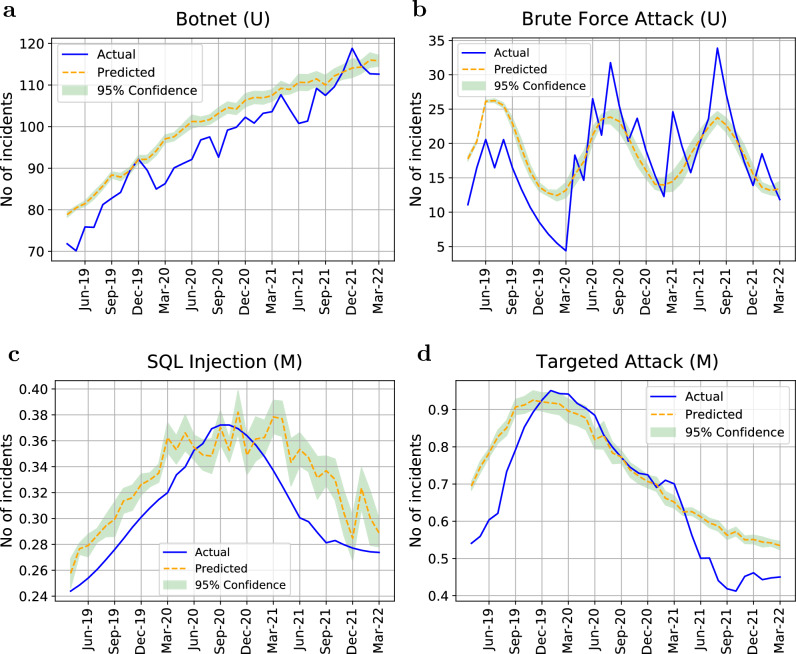
The B-LSTM validation results of predicting the number of attacks from April, 2019 to March, 2022. (U) indicates an univariate model while (M) indicates a multivariate model. (**a**) Botnet attack with M-SMAPE=0.03. (**b**) Brute force attack with M-SMAPE=0.13. (**c**) SQL injection attack with M-SMAPE=0.04 using the feature of NoM. (**d**) Targeted attack with M-SMAPE=0.06 using the feature of NoM. Y axis is normalised in the case of multivariate models to account for the different ranges of feature values.

The results of our model’s validation are provided in Fig. 3 and Table 5. As shown in Fig. 3, the predicted data points are well aligned with the ground truth. Our models successfully predicted the next 36 months of all the attacks’ trends with an average M-SMAPE of 0.25. Table 5 summarises the validation results of univariate and multivariate approaches using B-LSTM. The results show that with approximately 69% of all the attack types, the multivariate approach outperformed the univariate approach. As seen in Fig. 3, the threats that have a consistent increasing or emerging trend seemed to be more suitable for the univariate approach, while threats that have a fluctuating or decreasing trend showed less validation error when using the multivariate approach. The feature of ACA resulted in the best model for 33% of all the attack types, which makes it among the three most informative features that can boost the prediction performance. The PH accounts for 17% of all the attacks followed by NoM that accounts for 12%.

We additionally compared the performance of the proposed model B-LSTM with other models namely LSTM and ARIMA. The comparison covers the univariate and multivariate approaches of LSTM and B-LSTM, with two features in the case of multivariate approach namely NoI and NoM. The comparison is in terms of the Mean Absolute Percentage Error (MAPE) when predicting four common attack types, namely DDoS, Password Attack, Malware, and Ransomware. A comparison table is provided in Supplementary Table S3. The results illustrate the superiority of the B-LSTM model for most of the attack types.

### Trends analysis

**Figure 4:**
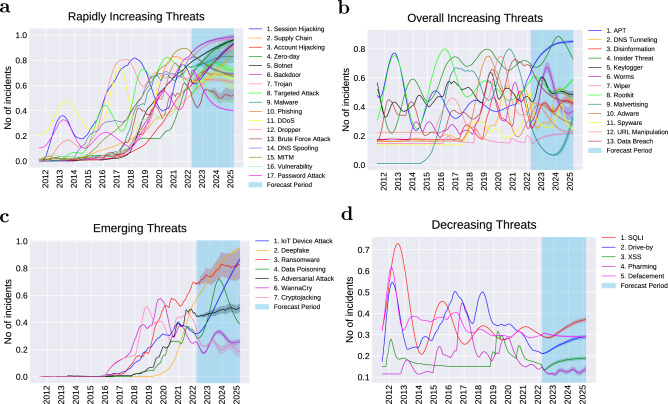
A bird’s eye view of threat trend categories. The period of the trend plots is between July, 2011 and March, 2025, with the period between April, 2022 and March, 2025 forecasted using B-LSTM. (**a**) Among rapidly increasing threats, as observed in the forecast period, some threats are predicted to continue a sharp increase until 2025 while others will probably level off. (**b**) Threats under this category have overall been increasing while fluctuating over the past 11 years. Recently, some of the overall increasing threats slightly declined however many of those are likely to recover and level off by 2025. (**c**) Emerging threats that began to appear and grow sharply after the year 2016, and are expected to continue growing at this increasing rate, while others are likely to slow down or stabilise by 2025. (**d**) Decreasing threats that peaked in the earlier years and have slowly been declining since then. This decreasing group are likely to level off however probably will not disappear in the coming 3 years. The Y axis is normalised to account for the different ranges of values across different attacks. The 95% confidence interval is shown for each threat prediction.

The forecast of each attack trend until the end of the first quarter of 2025 is given in Supplementary Figs. S1–S4. By visualising the historical data of each attack as well as the prediction for the next three years, we were able to analyse the overall trend of each attack. The attacks generally follow 4 types of trends: (1) rapidly increasing, (2) overall increasing, (3) emerging and (4) decreasing. The names of attacks for each category are provided in Fig. 4.

The first trend category is the rapidly increasing trend (Fig. 4a—approximately 40% of the attacks belong to this trend. We can see that the attacks belonging to this category have increased dramatically over the past 11 years. Based on the model’s prediction, some of these attacks will exhibit a steep growth until 2025. Examples include session hijacking, supply chain, account hijacking, zero-day and botnet. Some of the attacks under this category have reached their peak, have recently started stabilising, and will probably remain steady over the next 3 years. Examples include malware, targeted attack, dropper and brute force attack. Some attacks in this category, after a recent increase, are likely to level off in the next coming years. These are password attack, DNS spoofing and vulnerability-related attacks.

The second trend category is the overall increasing trend as seen in Fig. 4b. Approximately 31% of the attacks seem to follow this trend. The attacks under this category have a slower rate of increase over the years compared to the attacks in the first category, with occasional fluctuations as can be observed in the figure. Although some of the attacks show a slight recent decline (*e.g.*, malvertising, keylogger and URL manipulation), malvertising and keylogger are likely to recover and return to a steady state while URL manipulation is projected to continue a smooth decline. Other attacks typical of “cold” cyber-warfare like Advanced Persistent Threats (APT) and rootkits are already recovering from a small drop and will likely to rise to a steady state by 2025. Spyware and data breach have already reached their peak and are predicted to decline in the near future.

Next is the emerging trend as shown in Fig. 4c. These are the attacks that started to grow significantly after the year 2016, although many of them existed much earlier. In our study, around 17% of the attacks follow this trend. Some attacks have been growing steeply and are predicted to continue this trend until 2025. These are Internet of Things (IoT) device attack and deepfake. Other attacks have also been increasing rapidly since 2016, however, are likely to slow down after 2022. These include ransomware and adversarial attacks. Interestingly, some attacks that emerged after 2016 have already reached the peak and recently started a slight decline (*e.g.*, cryptojacking and WannaCry ransomware attack). It is likely that WannaCry will become relatively steady in the coming years, however, cryptojacking will probably continue to decline until 2025 thanks to the rise of proof-of-stake consensus mechanisms^53^.

The fourth and last trend category is the decreasing trend (Fig. 4d—only 12% of the attacks follow this trend. Some attacks in this category peaked around 2012, and have been slowly decreasing since then (*e.g.*, SQL Injection and defacement). The drive-by attack also peaked in 2012, however, had other local peaks in 2016 and 2018, after which it declined noticeably. Cross-site scripting (XSS) and pharming had their peak more recently compared to the other attacks, however, have been smoothly declining since then. All the attacks under this category are predicted to become relatively stable from 2023 onward, however, they are unlikely to disappear in the next 3 years.

### The threat cycle

**Figure 5:**
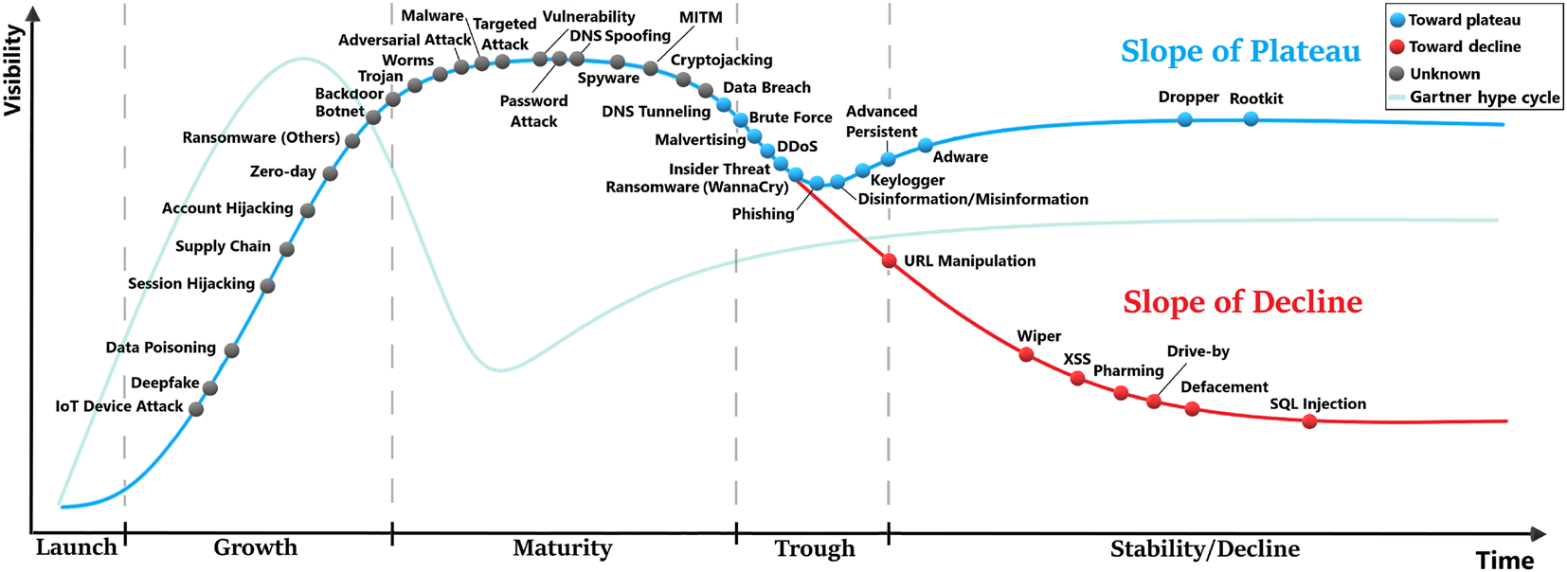
The threat cycle (TTC). The attacks go through 5 stages, namely, launch, growth, maturity trough, and stability/decline. A standard Gartner hype cycle (GHC) is shown with a vanishing green colour for a comparison to TTC. Both GHC and TTC have a peak, however, TTC’s peak is much wider with a slightly less steep curve during the growth stage. Some attacks in TTC do not recover after the trough and slide into the slope of decline. TTC captures the state of each attack in 2022, where the colour of each attack indicates which slope it would follow (e.g., plateau or decreasing) based on the predictive results until 2025. Within the trough stage, the attacks (in blue dot) are likely to arrive at the slope of plateau by 2025. The attacks (in red dot) will probably be on the slope of decline by 2025. The attacks with unknown final destination are coloured in grey.

This large-scale analysis involving the historical data and the predictions for the next three years enables us to come up with a generalisable model that traces the evolution and adoption of the threats as they pass through successive stages. These stages are named by the launch, growth, maturity, trough and stability/decline. We refer to this model as The Threat Cycle (or TTC), which is depicted in Fig. 5. In the launch phase, few incidents start appearing for a short period. This is followed by a sharp increase in terms of the number of incidents, growth and visibility as more and more cyber actors learn and adopt this new attack. Usually, the attacks in the launch phase are likely to have many variants as observed in the case of the WannaCry attack in 2017. At some point, the number of incidents reaches a peak where the attack enters the maturity phase, and the curve becomes steady for a while. Via the trough (when the attack experiences a slight decline as new security measures seem to be very effective), some attacks recover and adapt to the security defences, entering the slope of plateau, while others continue to smoothly decline although they do not completely disappear (*i.e.*, slope of decline). It is worth noting that the speed of transition between the different phases may vary significantly between the attacks.

As seen in Fig. 5, the attacks are placed on the cycle based on the slope of their current trend, while considering their historical trend and prediction. In the trough phase, we can see that the attacks will either follow the slope of plateau or the slope of decline. Based on the predicted trend in the blue zone in Fig. 4, we were able to indicate the future direction for some of the attacks close to the split point of the trough using different colours (blue or red). Brute force, malvertising, the Distributed Denial-of-Service attack (DDoS), insider threat, WannaCry and phishing are denoted in blue meaning that these are likely on their way to the slope of plateau. In the first three phases, it is usually unclear and difficult to predict whether a particular attack will reach the plateau or decline, thus, denoted in grey.

There are some similarities and differences between TTC and the well-known Gartner hype cycle (GHC)^54^. A standard GHC is shown in a vanishing green colour in Fig. 5. As TTC is specific to cyber threats, it has a much wider peak compared to GHC. Although both GHC and TTC have a trough phase, the threats decline slightly (while significant drop in GHC) as they exit their maturity phase, after which they recover and move to stability (slope of plateau) or decline.

Many of the attacks in the emerging category are observed in the growth phase. These include IoT device attack, deepfake and data poisoning. While ransomwares (except WannaCry) are in the growth phase, WannaCry already reached the trough, and is predicted to follow the slope of plateau. Adversarial attack has just entered the maturity stage, and cryptojacking is about to enter the trough. Although adversarial attack is generally regarded as a growing threat, interestingly, this machine-based prediction and introspection shows that it is maturing. The majority of the rapidly increasing threats are either in the growth or in the maturity phase. The attacks in the growth phase include session hijacking, supply chain, account hijacking, zero-day and botnet. The attacks in the maturity phase include malware, targeted attack, vulnerability-related attacks and Man-In-The-Middle attack (MITM). Some rapidly increasing attacks such as phishing, brute force, and DDoS are in the trough and are predicted to enter the stability. We also observe that most of the attacks in the category of overall increasing threats have passed the growth phase and are mostly branching to the slope of plateau or the slope of decline, while few are still in the maturity phase (*e.g.*, spyware). All of the decreasing threats are on the slope of decline. These include XSS, pharming, drive-by, defacement and SQL injection.

## Discussion

### Highlights and limitations

This study presents the development of a ML-based proactive approach for long-term prediction of cyber-attacks offering the ability to communicate effectively with the potential attacks and the relevant security measures in an early stage to plan for the future. This approach can contribute to the prevention of an incident by allowing more time to develop optimal defensive actions/tools in a contested cyberspace. Proactive approaches can also effectively reduce uncertainty when prioritising existing security measures or initiating new security solutions. We argue that cyber-security agencies should prioritise their resources to provide the best possible support in preventing fastest-growing attacks that appear in the launch phase of TTC or the attacks in the categories of the rapidly increasing or emerging trend as in Fig. 4a and c based on the predictions in the coming years.

In addition, our fully automated approach is promising to overcome the well-known issues of human-based analysis, above all expertise scarcity. Given the absence of the possibility of analysing with human’s subjective bias while following a purely quantitative procedure and data, the resulting predictions are expected to have lower degree of subjectivity, leading to consistencies within the subject. By fully automating this analytic process, the results are reproducible and can potentially be explainable with help of the recent advancements in Explainable Artificial Intelligence.

Thanks to the massive data volume and wide geographic coverage of the data sources we utilised, this study covers every facet of today’s cyber-attack scenario. Our holistic approach performs the long-term prediction on the scale of 36 countries, and is not confined to a specific region. Indeed, cyberspace is limitless, and a cyber-attack on critical infrastructure in one country can affect the continent as a whole or even globally. We argue that our Threat Cycle (TTC) provides a sound basis to awareness of and investment in new security measures that could prevent attacks from taking place. We believe that our tool can enable a collective defence effort by sharing the long-term predictions and trend analysis generated via quantitative processes and data and furthering the analysis of its regional and global impacts.

Zero-day attacks exploit a previously unknown vulnerability before the developer has had a chance to release a patch or fix for the problem^55^. Zero-day attacks are particularly dangerous because they can be used to target even the most secure systems and go undetected for extended periods of time. As a result, these attacks can cause significant damage to an organisation’s reputation, financial well-being, and customer trust. Our approach takes the existing research on using ML in the field of zero-day attacks to another level, offering a more proactive solution. By leveraging the power of deep neural networks to analyse complex, high-dimensional data, our approach can help agencies to prepare ahead of time, in-order to prevent the zero-day attack from happening at the first place, a problem that there is no existing fix for it despite our ability to detect it. Our results in Fig. 4a suggest that zero-day attack is likely to continue a steep growth until 2025. If we know this information, we can proactively invest on solutions to prevent it or slow down its rise in the future, since after all, the ML detection approaches may not be alone sufficient to reduce its effect.

A limitation of our approach is its reliance on a restricted dataset that encompasses data since 2011 only. This is due to the challenges we encountered in accessing confidential and sensitive information. Extending the prediction phase requires the model to make predictions further into the future, where there may be more variability and uncertainty. This could lead to a decrease in prediction accuracy, especially if the underlying data patterns change over time or if there are unforeseen external factors that affect the data. While not always the case, this uncertainty is highlighted by the results of the Bayesian model itself as it expresses this uncertainty through the increase of the confidence interval over time (Fig. 3a and b). Despite incorporating the Bayesian model to tackle the epistemic uncertainty, our model could benefit substantially from additional data to acquire a comprehensive understanding of past patterns, ultimately improving its capacity to forecast long-term trends. Moreover, an augmented dataset would allow ample opportunity for testing, providing greater confidence in the model’s resilience and capability to generalise.

Further enhancements can be made to the dataset by including pivotal dates (such as anniversaries of political events and war declarations) as a feature, specifically those that experience a high frequency of cyber-attacks. Additionally, augmenting the dataset with digital traces that reflect the attackers’ intentions and motivations obtained from the dark web would be valuable. Other informative features could facilitate short-term prediction, specifically to forecast the on-set of each attack.

### Future work

Moving forward, future research can focus on augmenting the dataset with additional samples and informative features to enhance the model’s performance and its ability to forecast the trend in the longer-term. Also, the work opens a new area of research that focuses on prognosticating the disparity between the trend of cyber-attacks and the associated technological solutions and other variables, with the aim of guiding research investment decisions. Subsequently, TTC could be improved by adopting another curve model that can visualise the current development of relevant security measures. The threat trend categories (Fig. 4) and TTC (Fig. 5) show how attacks will be visible in the next three years and more, however, we do not know where the relevant security measures will be. For example, data poisoning is an AI-targeted adversarial attack that attempts to manipulate the training dataset to control the prediction behaviour of a machine-learned model. From the scientific literature data (*e.g.*, Scopus), we could analyse the published articles studying the data poisoning problem and identify the relevant keywords of these articles (*e.g.*, Reject on Negative Impact (RONI) and Probability of Sufficiency (PS)). RONI and PS are typical methods used for detecting poisonous data by evaluating the effect of individual data points on the performance of the trained model. Likewise, the features that are informative, discriminating or uncertainty-reducing for knowing how the relevant security measures evolve exist within such online sources in the form of author’s keywords, number of citations, research funding, number of publications, *etc*.

**Table 1:** Literature review summary.

References	Problem domain	Detection/prediction	Forecast period	Forecast coverage	Methods
^14^	Detect different types of attacks	Detection	N/A	N/A	Feature extraction, deep reinforcement learning
^15^	Malicious traffic detection	Detection	N/A	N/A	Deep neural network with attention mechanism
^16,17^	Forecast attack count	Prediction	1–7 days	Multiple targets	ARIMA model
^18^	Forecast attack count	Prediction	Months	Organisation	Unconventional signals, lagged feature selection, concept drift training
^19^	Forecast attack motivation and opportunity	Prediction	1 week	1 target	Social media analysis, SVM, CNN
^20^	Forecast attack count	Prediction	1 week or month	Organisation	Digital traces, ARIMA, ARIMAX, LSTM
^21^	Predict next attack in the chain	Prediction	N/A	1 target	Bayesian network
^22^	Predict intrusion detection alerts	Prediction	Minutes or hours	Organisation	Stream processing, sequential rule mining
^23^	Forecast if a data breach will occur	Prediction	Months	Organisation	Externally measurable features, Random Forest
^24^	Reconnaissance detection	Detection	N/A	N/A	LSTM, CNN
^25^	Forecast if a machine will be infected	Prediction	Months	Machine	Binary file analysis, semi-supervised learning
^26^	Forecast if an IP address will attack	Prediction	24 hours	N/A	Entity reputation and scoring, decision trees

**Table 2:** Hackmageddon data - entry example.

Date	Description	Attack	Target country
31/07/2019	Gadsden independent school district is hit by a ransomware attack.	Malware	US

**Table 3:** Transformed data - entry example with dummy values.

Month	Malware US	Malware UK	.	Malware all	DDoS US	DDoS UK	.	DDoS all
July 2019	50	40	.	1000	20	15	.	100

“All” refers to all countries involved in the study. Columns are repeated for each pair of attack and country, with a total of 42 attacks in 36 countries.

**Table 4:** Additional data features with dummy values.

Month	NoM malware	NoM ransomware	ACA US	ACA All	PH UK	PH All
July 2019	500	400	12,000	100,000	8	400

NoM stands for the number of attack mentions in the scientific literature. ACA stands for the number of tweets related to armed conflict areas/wars. PH stands for the number of public holidays. “All” refers to all countries involved in the study. Some features are not shown to keep the table at reasonable width. More specifically, the number of mentions in the actual data is recorded for 42 attacks, and the number of conflicts and holidays are recorded for 36 countries in the actual table.

**Table 5:** The validation results of univariate and multivariate approach using B-LSTM to forecast 42 attacks next 36 months.

Attack	M-SMAPE (univariate)	M-SMAPE (multivariate)	Best features (multivariate)
Adware	0.35	0.29	ACA
Backdoor	0.10	0.03	ACA
Cryptojacking	0.40	0.34	ACA
Data Poisoning	0.47	0.46	ACA
Defacement	0.36	0.06	ACA
DNS Tunneling	0.48	0.42	ACA
Keylogger	0.17	0.14	ACA
Pharming	0.59	0.27	ACA
Trojan	0.31	0.30	ACA
Vulnerability	0.33	0.25	ACA
WannaCry	0.58	0.57	ACA
Wiper	0.43	0.14	ACA
Worms	0.50	0.37	ACA
XSS	0.47	0.17	ACA
Advanced Persistent	0.84	0.32	PH
DNS Spoofing	0.48	0.36	PH
Drive-by	0.46	0.27	PH
Insider Threat	0.17	0.07	PH
Malvertising	0.38	0.25	PH
Session Hijacking	0.39	0.34	PH
URL manipulation	0.47	0.36	PH
Data Breach	0.27	0.24	NoM
Disinformation	0.45	0.36	NoM
Phishing	0.22	0.21	NoM
SQL Injection	0.53	0.06	NoM
Targeted Attack	0.25	0.22	NoM
Password Attack	0.59	0.52	NoM, ACA, PH
Rootkit	0.19	0.15	NoM, ACA, PH
Spyware	0.63	0.48	NoM, ACA, PH
Account Hijacking	0.09	0.49	ACA
Adversarial Attack	0.37	0.63	NoM, ACA, PH
Botnet	0.03	0.17	PH
Brute Force Attack	0.13	0.28	ACA
DDoS	0.22	0.23	PH
Deepfake	0.17	0.52	PH
Dropper	0.12	0.37	PH
IoT device attack	0.16	0.21	PH
Malware	0.12	0.27	PH
MITM	0.14	0.32	PH
Ransomware	0.26	0.53	NoM
Supply chain	0.15	0.33	PH
Zero-day	0.30	0.63	NoM

For each attack, the best feature(s) when using the multivariate model are displayed in the last column. NoM stands for the number of attack mentions in the scientific literature. ACA stands for the number of tweets related to armed conflict areas/wars. PH stands for the number of public holidays.

## Supplementary Information

Supplementary Information.

## Data Availability

As requested by the journal, the data used in this paper is available to editors and reviewers upon request. The data will be made publicly available and can be accessed at the following link after the paper is published. https://github.com/zaidalmahmoud/Cyber-threat-forecast.

## References

[1] Ghafur S (2019). A retrospective impact analysis of the wannacry cyberattack on the NHS. NPJ Digit. Med..

[2] Alrzini JRS, Pennington D (2020). A review of polymorphic malware detection techniques. Int. J. Adv. Res. Eng. Technol..

[3] Lazarevic, A., Ertoz, L., Kumar, V., Ozgur, A. & Srivastava, J. A comparative study of anomaly detection schemes in network intrusion detection. In: *Proceedings of the 2003 SIAM International Conference on Data Mining*, 25–36 (SIAM, 2003).

[4] Kebir O, Nouaouri I, Rejeb L, Said LB (2022). Atipreta: An analytical model for time-dependent prediction of terrorist attacks. Int. J. Appl. Math. Comput. Sci..

[5] Anticipating cyber attacks: There’s no abbottabad in cyber space. *Infosecurity Magazine* https://www.infosecurity-magazine.com/white-papers/anticipating-cyber-attacks (2015).

[6] Jumper J (2021). Highly accurate protein structure prediction with alphafold. Nature.

[7] Baek M (2021). Accurate prediction of protein structures and interactions using a three-track neural network. Science.

[8] Gibney E (2022). Where is russia’s cyberwar? researchers decipher its strategy. Nature.

[9] Passeri, P. Hackmageddon data set. *Hackmageddon* https://www.hackmageddon.com (2022).

[10] Chen C-M (2023). A provably secure key transfer protocol for the fog-enabled social internet of vehicles based on a confidential computing environment. Veh. Commun..

[11] Nagasree Y (2023). Preserving privacy of classified authentic satellite lane imagery using proxy re-encryption and UAV technologies. Drones.

[12] Kavitha A (2022). Security in IoT mesh networks based on trust similarity. IEEE Access.

[13] Salih, A., Zeebaree, S. T., Ameen, S., Alkhyyat, A. & Shukur, H. M A survey on the role of artificial intelligence, machine learning and deep learning for cybersecurity attack detection. In: *2021 7th International Engineering Conference “Research and Innovation amid Global Pandemic” (IEC)*, 61–66 (IEEE, 2021).

[14] Ren K, Zeng Y, Cao Z, Zhang Y (2022). Id-rdrl: A deep reinforcement learning-based feature selection intrusion detection model. Sci. Rep..

[15] Liu X, Liu J (2021). Malicious traffic detection combined deep neural network with hierarchical attention mechanism. Sci. Rep..

[16] Werner, G., Yang, S. & McConky, K. Time series forecasting of cyber attack intensity. In *Proceedings of the 12th Annual Conference on Cyber and Information Security Research*, 1–3 (2017).

[17] Werner, G., Yang, S. & McConky, K. Leveraging intra-day temporal variations to predict daily cyberattack activity. In *2018 IEEE International Conference on Intelligence and Security Informatics (ISI)*, 58–63 (IEEE, 2018).

[18] Okutan, A., Yang, S. J., McConky, K. & Werner, G. Capture: cyberattack forecasting using non-stationary features with time lags. In *2019 IEEE Conference on Communications and Network Security (CNS)*, 205–213 (IEEE, 2019).

[19] Munkhdorj B, Yuji S (2017). Cyber attack prediction using social data analysis. J. High Speed Netw..

[20] Goyal, P. *et al.* Discovering signals from web sources to predict cyber attacks. arXiv preprint arXiv:1806.03342 (2018).

[21] Qin, X. & Lee, W. Attack plan recognition and prediction using causal networks. In *20th Annual Computer Security Applications Conference*, 370–379 (IEEE, 2004).

[22] Husák, M. & Kašpar, J. Aida framework: real-time correlation and prediction of intrusion detection alerts. In: *Proceedings of the 14th international conference on availability, reliability and security*, 1–8 (2019).

[23] Liu, Y. *et al.* Cloudy with a chance of breach: Forecasting cyber security incidents. In: *24th USENIX Security Symposium (USENIX Security 15)*, 1009–1024 (2015).

[24] Malik J (2020). Hybrid deep learning: An efficient reconnaissance and surveillance detection mechanism in sdn. IEEE Access.

[25] Bilge, L., Han, Y. & Dell’Amico, M. Riskteller: Predicting the risk of cyber incidents. In *Proceedings of the 2017 ACM SIGSAC Conference on Computer and Communications Security*, 1299–1311 (2017).

[26] Husák M, Bartoš V, Sokol P, Gajdoš A (2021). Predictive methods in cyber defense: Current experience and research challenges. Futur. Gener. Comput. Syst..

[27] Stephens G (2008). Cybercrime in the year 2025. Futurist.

[28] Adamov, A. & Carlsson, A. The state of ransomware. Trends and mitigation techniques. In *EWDTS*, 1–8 (2017).

[29] Shoufan A, Damiani E (2017). On inter-rater reliability of information security experts. J. Inf. Secur. Appl..

[30] Cha Y-O, Hao Y (2022). The dawn of metamaterial engineering predicted via hyperdimensional keyword pool and memory learning. Adv. Opt. Mater..

[31] Elsevier research products apis. *Elsevier Developer Portal* https://dev.elsevier.com (2022).

[32] Twitter api v2. *Developer Platform* https://developer.twitter.com/en/docs/twitter-api (2022).

[33] holidays 0.15. *PyPI. The Python Package Index* https://pypi.org/project/holidays/ (2022).

[34] Visser M, van Eck NJ, Waltman L (2021). Large-scale comparison of bibliographic data sources: Scopus, web of science, dimensions, crossref, and microsoft academic. Quant. Sci. Stud..

[35] 2021 trends show increased globalized threat of ransomware. *Cybersecurity and Infrastructure Security Agency* https://www.cisa.gov/uscert/ncas/alerts/aa22-040a (2022).

[36] Lai, K. K., Yu, L., Wang, S. & Huang, W. Hybridizing exponential smoothing and neural network for financial time series predication. In *International Conference on Computational Science*, 493–500 (Springer, 2006).

[37] Huang, B., Ding, Q., Sun, G. & Li, H. Stock prediction based on Bayesian-lstm. In *Proceedings of the 2018 10th International Conference on Machine Learning and Computing*, 128–133 (2018).

[38] Mae Y, Kumagai W, Kanamori T (2021). Uncertainty propagation for dropout-based Bayesian neural networks. Neural Netw..

[39] Scopus preview. *Scopus* https://www.scopus.com/home.uri (2022).

[40] Jia, P., Chen, H., Zhang, L. & Han, D. Attention-lstm based prediction model for aircraft 4-d trajectory. *Sci. Rep.* **12** (2022). 10.1038/s41598-022-19794-1 PMC9478158 36109612

[41] Chandra R, Goyal S, Gupta R (2021). Evaluation of deep learning models for multi-step ahead time series prediction. IEEE Access.

[42] Gers FA, Schmidhuber J, Cummins F (2000). Learning to forget: Continual prediction with lstm. Neural Comput..

[43] Sagheer A, Kotb M (2019). Unsupervised pre-training of a deep lstm-based stacked autoencoder for multivariate time series forecasting problems. Sci. Rep..

[44] Swiler, L. P., Paez, T. L. & Mayes, R. L. Epistemic uncertainty quantification tutorial. In *Proceedings of the 27th International Modal Analysis Conference* (2009).

[45] Gal, Y. & Ghahramani, Z. Dropout as a bayesian approximation: Representing model uncertainty in deep learning. arXiv preprint arXiv:1506.02142v6 (2016).

[46] Chollet, F. *Deep Learning with Python*, 2 edn. (Manning Publications, 2017).

[47] Xu, J., Li, Z., Du, B., Zhang, M. & Liu, J. Reluplex made more practical: Leaky relu. In *2020 IEEE Symposium on Computers and Communications (ISCC)*, 1–7 (IEEE, 2020).

[48] Gal, Y., Hron, J. & Kendall, A. Concrete dropout. *Adv. Neural Inf. Process. Syst.* **30** (2017).

[49] Shcherbakov MV (2013). A survey of forecast error measures. World Appl. Sci. J..

[50] Bergstra, J. & Bengio, Y. Random search for hyper-parameter optimization. *J. Mach. Learn. Res.* **13** (2012).

[51] Kingma, D. P. & Ba, J. Adam: A method for stochastic optimization. arXiv preprint arXiv:1412.6980 (2014).

[52] Krizhevsky A, Sutskever I, Hinton GE (2017). Imagenet classification with deep convolutional neural networks. Commun. ACM.

[53] Shifferaw Y, Lemma S (2021). Limitations of proof of stake algorithm in blockchain: A review. Zede J..

[54] Dedehayir O, Steinert M (2016). The hype cycle model: A review and future directions. Technol. Forecast. Soc. Chang..

[55] Abri, F., Siami-Namini, S., Khanghah, M. A., Soltani, F. M. & Namin, A. S. Can machine/deep learning classifiers detect zero-day malware with high accuracy?. In *2019 IEEE International Conference on Big Data (Big Data)*, 3252–3259 (IEEE, 2019).

