# Supplementary material for: A holistic and proactive approach to forecasting cyber threats
Source: Sci Rep. 2023 May 17;13:8049. doi: 10.1038/s41598-023-35198-1 (PMC10192224; doi:10.1038/s41598-023-35198-1)
Supplement: Supplementary file 1 — Supplementary Information. [file 41598_2023_35198_MOESM1_ESM.pdf]

A Holistic and Proactive Approach to Forecasting Cyber Threats

Zaid Almahmoud, Paul D. Yoo, Omar Alhussein, Ilyas Farhat and Ernesto Damiani

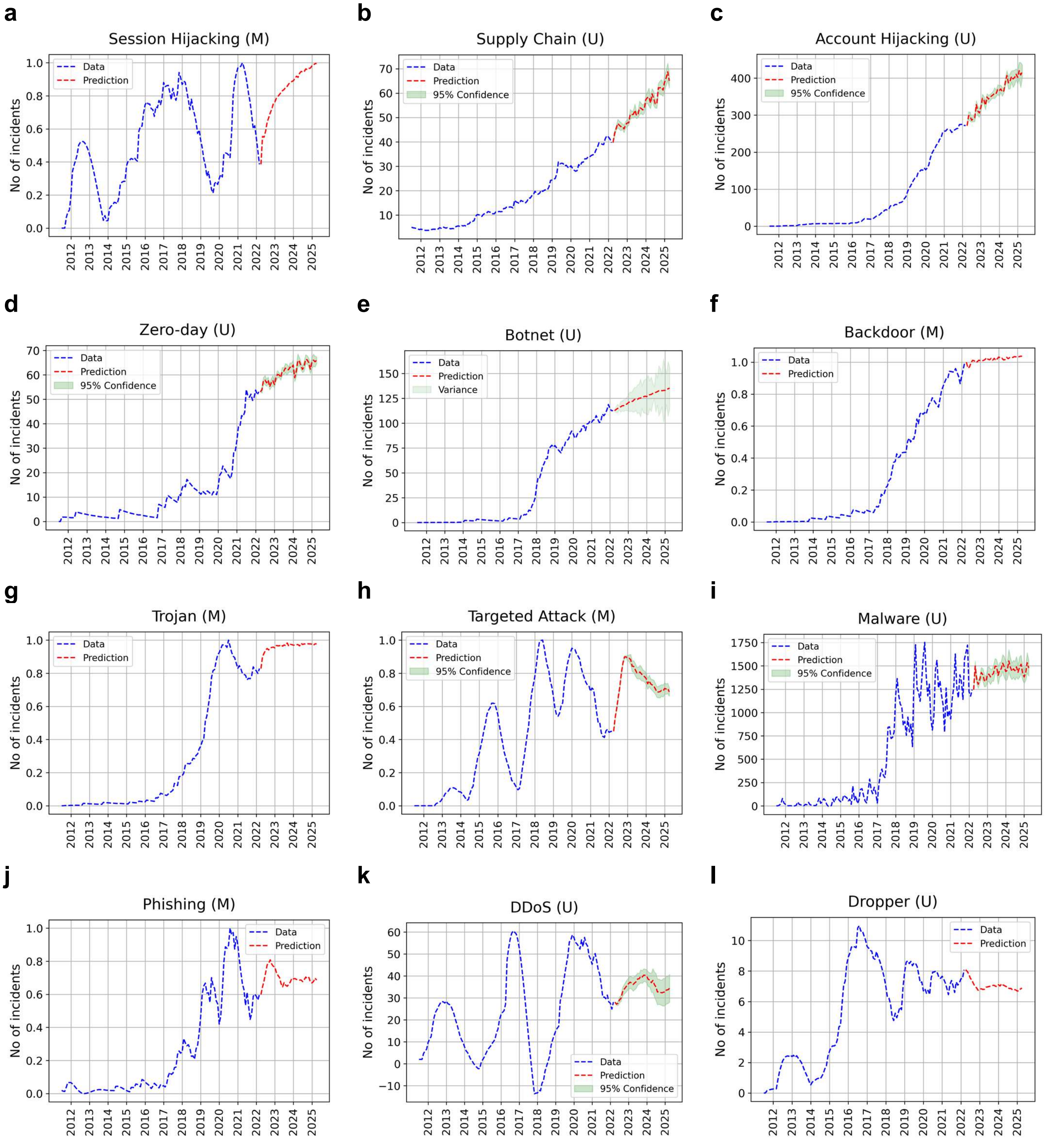

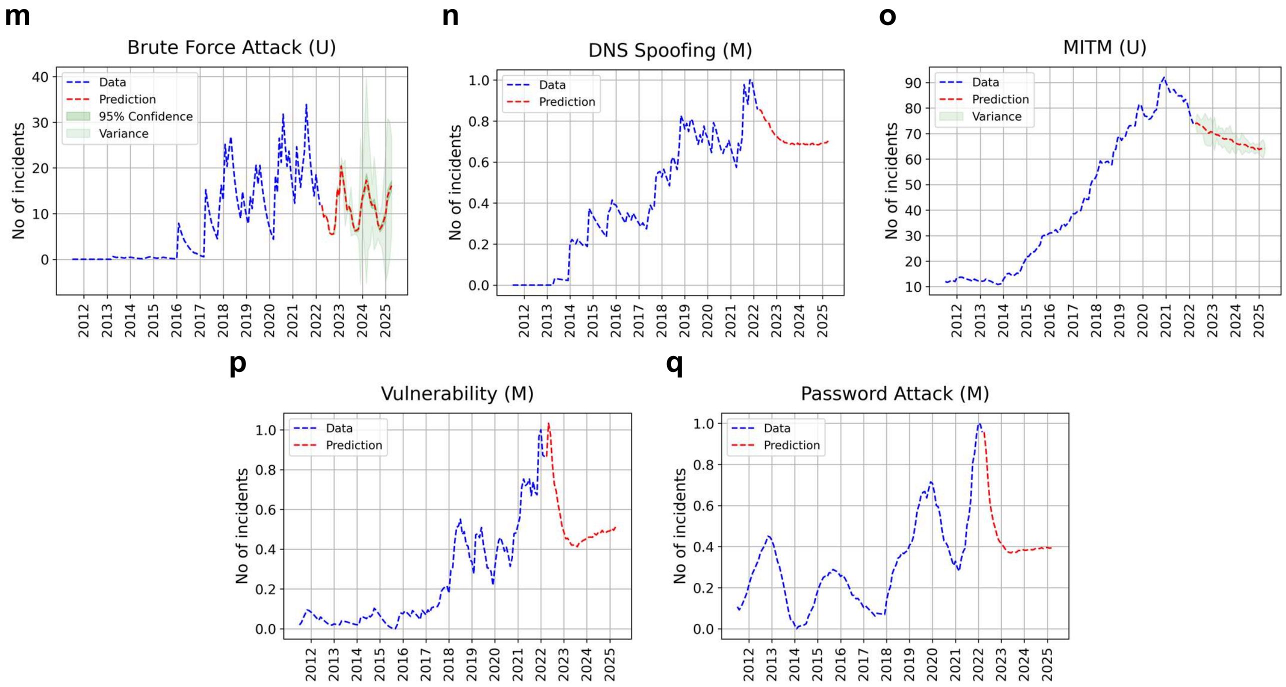

Supplementary Figure S1 | The prediction of rapidly increasing threats until March 2025 using Bayesian long short-term memory (B-LSTM). The figures are ordered by the steepness of the curve in the forecasted period. (U) indicates a univariate model while (M) indicates a multivariate model. The Y axis is normalised in the case of multivariate models to account for the different ranges of feature values. The figures show the 95% confidence interval and the variance of the predicted distribution while omitted when they are too small to be shown within the plot area.

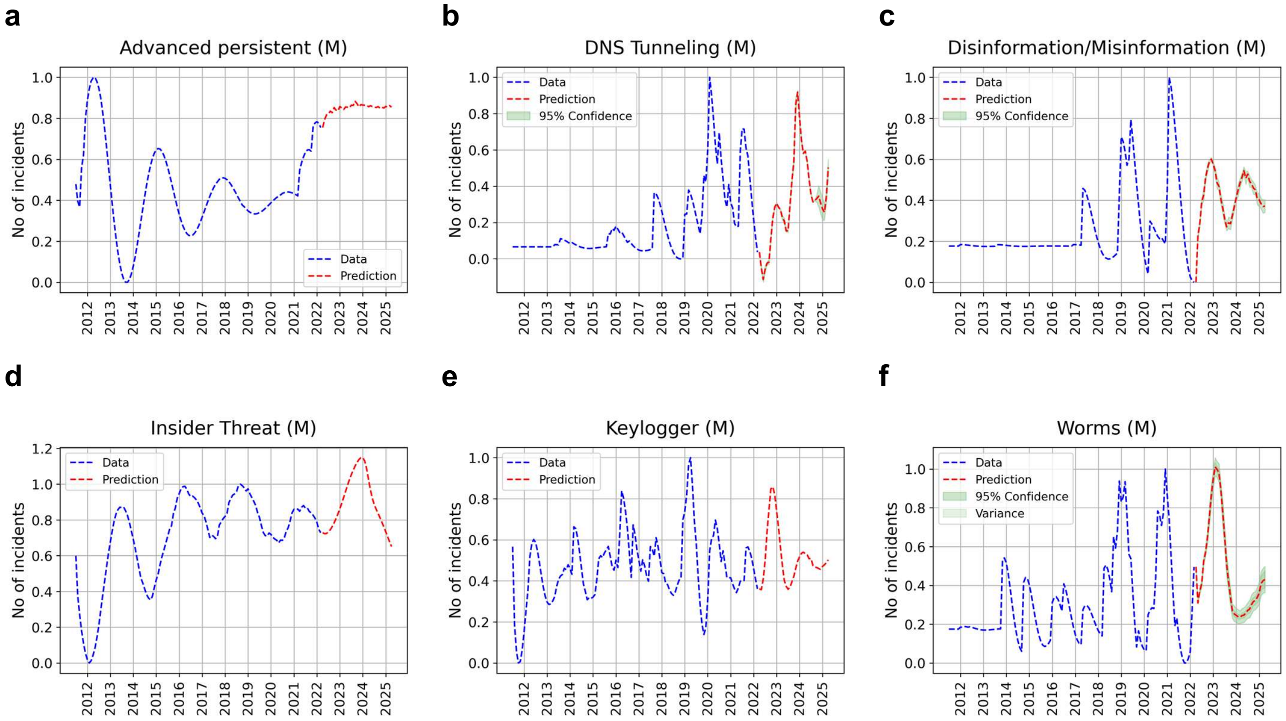

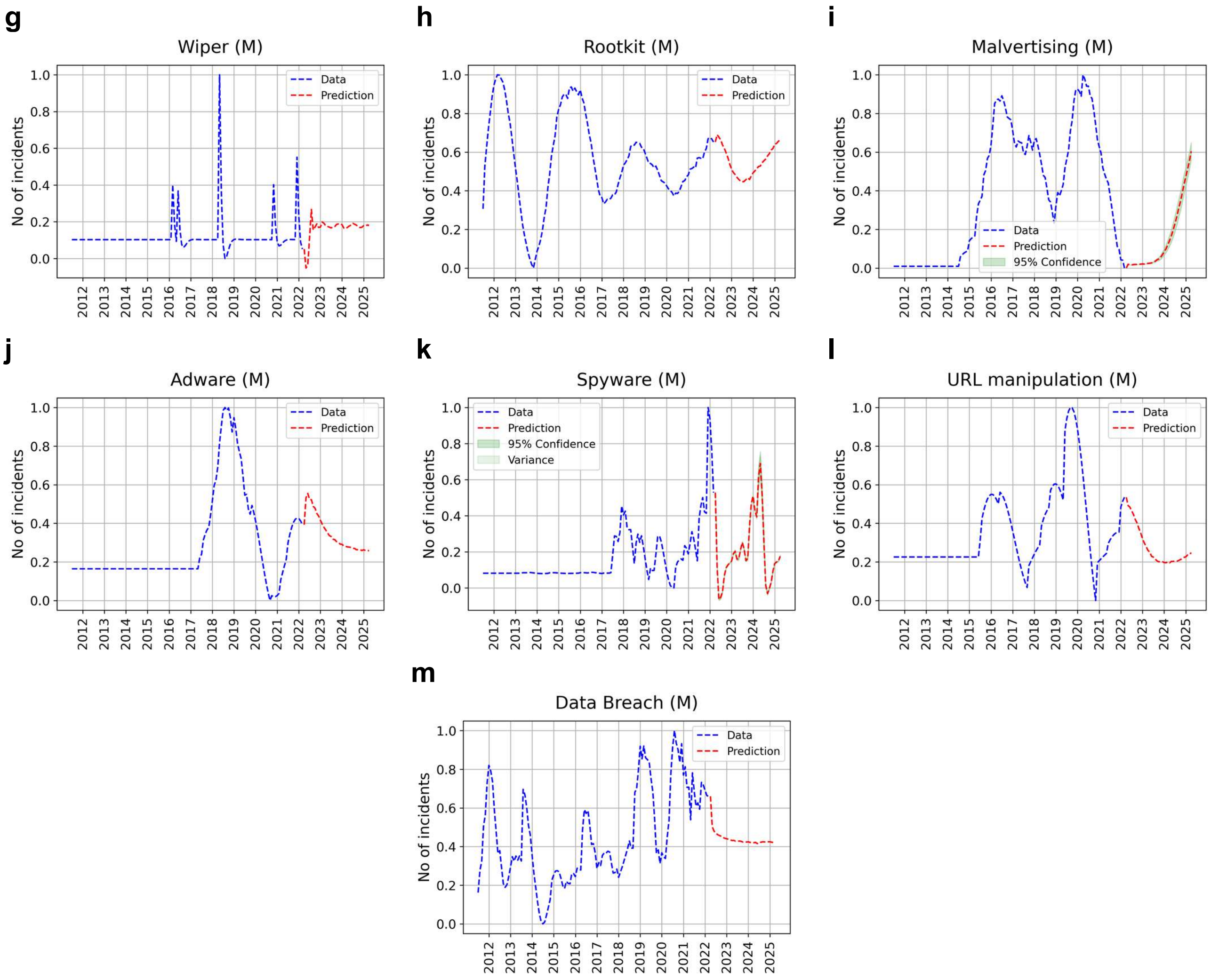

Supplementary Figure S2 | The prediction of overall increasing threats until March 2025 using Bayesian long short-term memory (B-LSTM). The figures are ordered by the steepness of the curve in the forecasted period. (M) indicates a multivariate model. The Y axis is normalised in the case of multivariate models to account for the different ranges of feature values. The figures show the 95% confidence interval and the variance of the predicted distribution while omitted when they are too small to be shown within the plot area.

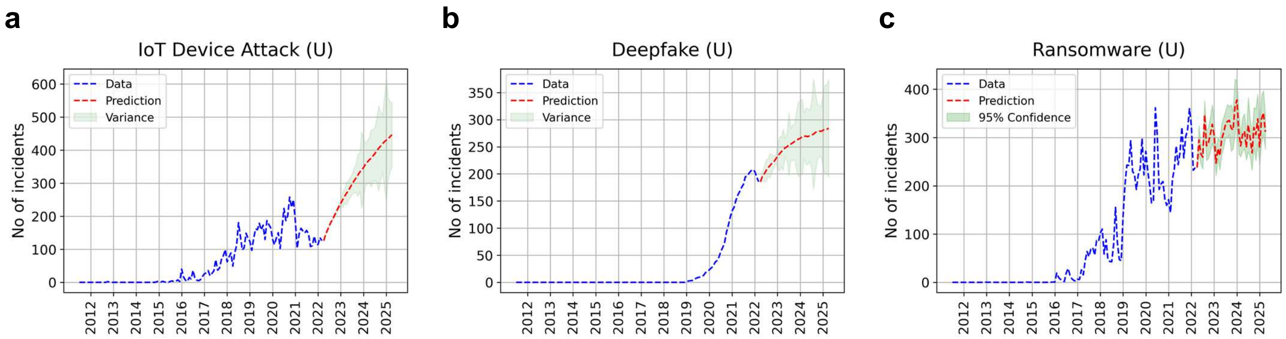

d e f

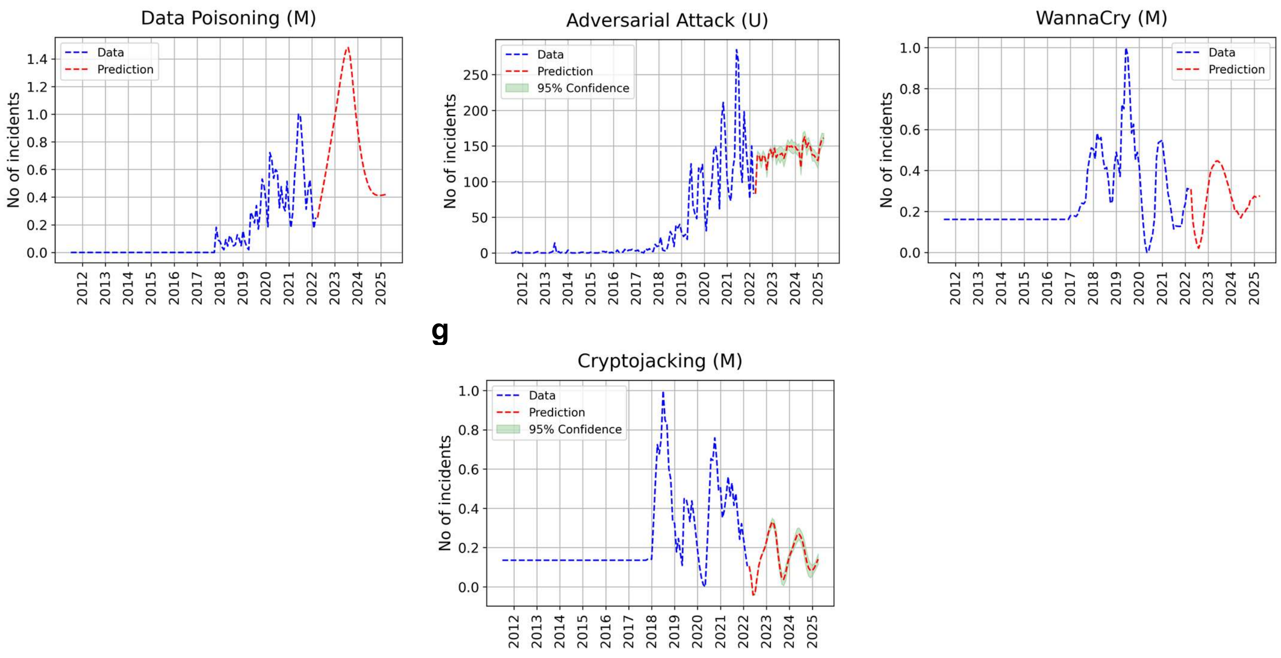

Supplementary Figure S3 | The prediction of emerging threats until March 2025 using Bayesian long short-term memory (B-LSTM). The figures are ordered by the steepness of the curve in the forecasted period. (U) indicates a univariate model while (M) indicates a multivariate model. The Y axis is normalised in the case of multivariate models to account for the different ranges of feature values. The figures show the 95% confidence interval and the variance of the predicted distribution while omitted when they are too small to be shown within the plot area.

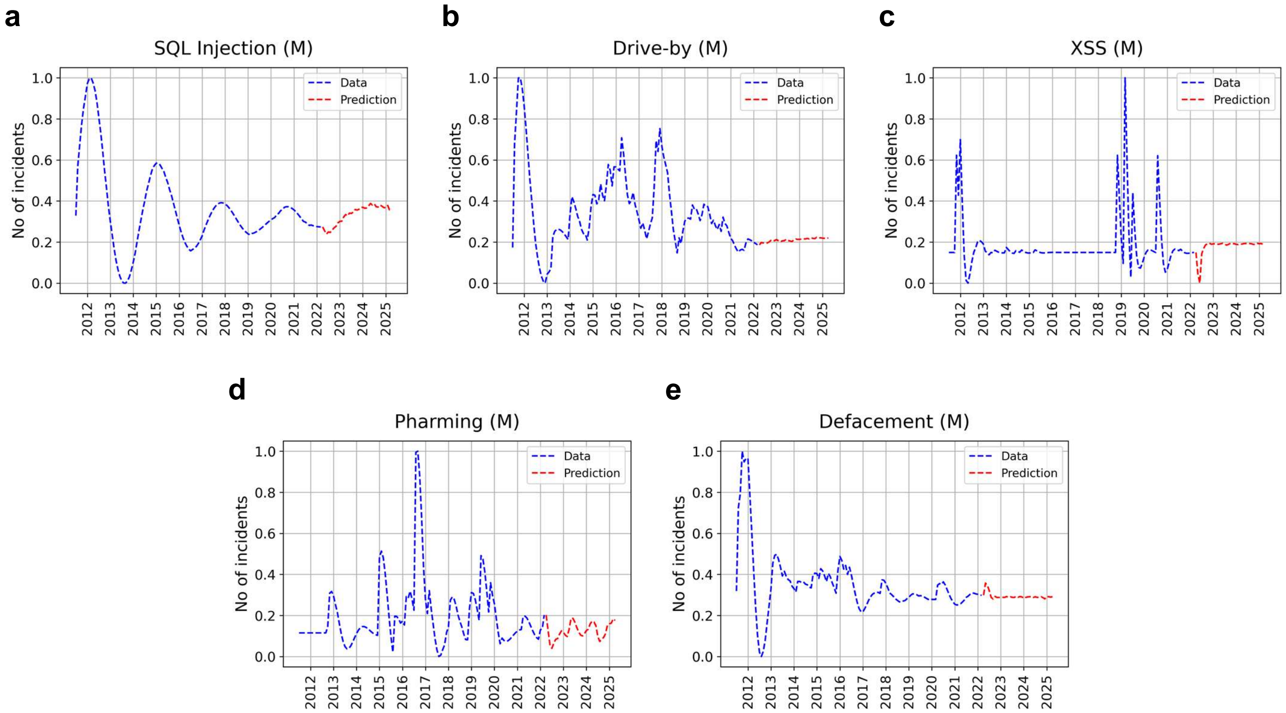

Supplementary Figure S4 | The prediction of decreasing threats until March 2025 using Bayesian long short-term memory (B-LSTM). (M) indicates a multivariate model. The Y axis is normalised in the case of multivariate models to account for the different ranges of feature values.

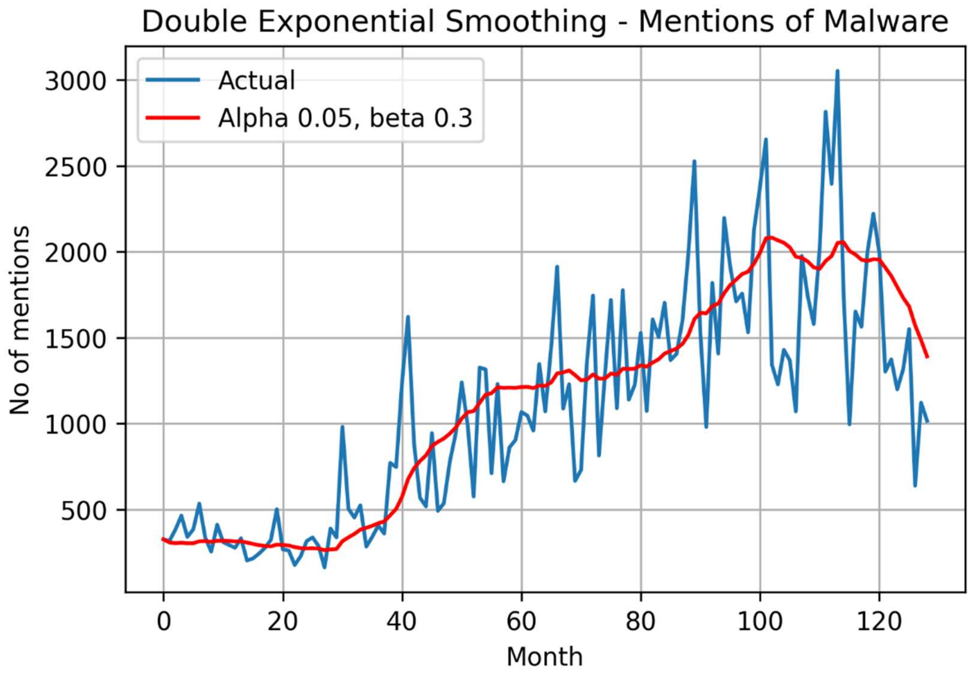

Supplementary Figure S5 | Double exponential smoothing (DES) for the number of mentions (NoM) of the malware attack in the scientific literature. The smoothing captures the trend to improve the prediction. The values of alpha and beta are chosen in a such way that the prediction error is minimised.

Supplementary Table S1 | Countries involved in the study

No.	Country Name	Country Code
1	United States of America	US
2	United Kingdom	GB
3	Canada	CA
4	Australia	AU
5	Ukraine	UA
6	Russia	RU
7	France	FR
8	Germany	DE
9	Brazil	BR
10	China	CN
11	Japan	JP
12	Pakistan	PK
13	North Korea	KP
14	South Korea	KR

15	India	IN
16	Taiwan	TW
17	Netherlands	NL
18	Spain	ES
19	Sweden	SE
20	Mexico	MX
21	Iran	IR
22	Israel	IL
23	Saudi Arabia	SA
24	Syria	SY
25	Finland	FI
26	Ireland	IE
27	Austria	AT
28	Norway	NO
29	Switzerland	CH
30	Italy	IT
31	Malaysia	MY
32	Egypt	EG
33	Turkey	TR
34	Portugal	PT
35	Palestine	PS
36	United Arab Emirates	AE

The country code is used in our dataset in the naming of the columns. The number of monthly attacks was quantified for each of the listed countries and for 42 attack types. The final prediction of threats was performed for all the countries combined.

Supplementary Table S2 | Keywords and phrases in the query for obtaining the feature Armed Conflict Areas/Wars (ACA)

	**Keyword 1**	**Keyword 2**	**Keyword 3**	**Keyword 4**
**Phrase 1**	$country_name$	WAR	MILITARY	
**Phrase 2**	$country_name$	WAR	ARMED	FORCE
**Phrase 3**	$country_name$	CONFLICT	POLITIC	
**Phrase 4**	$country_name$	MILITARY	ATTACK	
**Phrase 5**	$country_name$	ARMED	FORCE	ATTACK

The keywords are joined by logical AND operator and the phrases are joined by logical OR operator. A keyword between dollar signs indicates a variable. The query was repeated for the 36 countries in the study.

Supplementary Table S3 | Mean Absolute Percentage Error (MAPE) for 4 attacks and 5 models

**Attack/Model**	ARIMA	LSTM (U)	LSTM (M)	B-LSTM (U)	B-LSTM (M)
DDoS	1.43	0.63	0.93	0.65	0.53
Password Attack	0.60	0.69	0.70	0.56	0.72
Malware	0.61	2.88	1.96	5.47	1.11
Ransomware	0.85	0.37	1.04	0.36	0.59

(U) indicates a univariate model while (M) indicates a multivariate model.
